# Process Optimization for Manufacturing PAN-Based Conductive Yarn with Carbon Nanomaterials through Wet Spinning

**DOI:** 10.3390/polym13203544

**Published:** 2021-10-14

**Authors:** Hyelim Kim, Hyeongmin Moon, Daeyoung Lim, Wonyoung Jeong

**Affiliations:** Material and Component Convergence R&D Department, Korea Institute of Industrial Technology (KITECH), Ansan 15588, Gyeonggi-do, Korea; hyelim1221@kitech.re.kr (H.K.); hm9487@kitech.re.kr (H.M.); zoro1967@kitech.re.kr (D.L.)

**Keywords:** vapor grown carbon nanofiber, multi-wall carbon nanotube, hybrid carbon nanofiller, wet spinning, poly(acrylonitrile), conductive yarn, electrical property

## Abstract

This study aimed to manufacture PAN-based conductive yarn using a wet-spinning process. Two types of carbon nanomaterials, multiwall carbon nanotubes (MWCNT) and carbon nanofiber (CNF), were used alone or in a mixture. First, to derive the optimal composite solution condition for the wet spinning process, a composite solution was prepared with carbon nanomaterials of the same total mass weight (%) and three types of mechanical stirring were performed: mechanical stirring, ultra-sonication, and ball milling. A ball milling process was finally selected by analyzing the viscosity. Based on the above results, 8, 16, 24, and 32 wt% carbon nanomaterial/PAN composite solutions were prepared to produce wet spinning-based composite films before preparing a conductive yarn, and their physical and electrical properties were examined. By measuring the viscosity of the composite solution and the surface resistance of the composite film according to the type and content of carbon nanomaterials, a suitable range of viscosity was found from 10^3^ cP to 10^5^ cP, and the electrical percolation threshold was from 16 wt% carbon nanomaterial/PAN, which showed a surface resistance of 10^6^ Ω/sq or less. Wet spinning was possible with a PAN-based composite solution with a high content of carbon nanomaterials. The crystallinity, crystal orientation, tenacity, and thermal properties were improved when CNF was added up to 24 wt%. On the other hand, the properties deteriorated when CNTs were added alone due to aggregation. Mixing CNT and CNF resulted in poorer properties than with CNF alone, but superior properties to CNT alone. In particular, the electrical properties after incorporating 8 wt% CNT/16 wt% CNF into the PAN, 10^6^ Ω/cm was similar to the PAN-based conductive yarn containing 32 wt% CNF. Therefore, this yarn is expected to be applicable to various smart textiles and wearable devices because of its improved physical properties such as strength and conductivity.

## 1. Introduction

As the interest in smart textiles and wearable electronic devices is continuously increasing, a conductive yarn with excellent electrical conductivity and durability has been highlighted as an important factor for end-users. The shape of the conductive yarn is mainly wire, staple fiber, or multi-filaments, which can be obtained by coating and filling non-conductive fibers with carbon, silver, copper, aluminum, titanium, and stainless steel, which are conducting materials [1,2]. Among them, conductive yarn, which is manufactured by coating a conductive material such as silver, stainless steel, or copper on the spun yarn surface, can be harmful to the human body and show a decrease in conductivity due to peel-off of the coated particles after several uses [3,4]. Conductive polymers are presented as alternative materials for these coated fibers. However, conductive polymers such as PEDOT: PSS require an additional process of post treatment or the inclusion of additives. Therefore, it is time consuming, which limits the production of fibers in small batches, and is expensive [5]. Accordingly, research on conductive yarn based on spinning processes such as dry-jet spinning and wet spinning is steadily progressing by manufacturing a conductive nanofiller/polymer composite solution incorporating carbon nanofiller and metal nanowires in the polymer [3,6,7]. In particular, research on wet spinning for a carbon nanomaterial/polymer composite solution is in progress [8,9,10,11,12,13,14,15]. It is common to spinning a PAN polymer through wet spinning and does not require heat during the process. If the fiber needs additional characteristics by adding functional fillers, melt-blown cannot be an option for this because the fillers can be burnt during melt spinning. On the other hand, PAN fiber is easy to bear various fillers. PAN is a prominent material for both characterized and electric conductive fiber.

Wet spinning is the most suitable method to process PAN fibers because the polymer decomposes before it reaches the melting point so the poly-acrylonitrile is unable to be melt-blown. Among the wet spinning processes, dry-jet wet spinning is a dominant process for PAN fibers due to the alignment of polymer orientation, enhancing the physical properties. Unlike polymer dopes that are swallowed immediately in the coagulation bath during wet spinning, dry-jet wet spinning has an air gap before the dope solution is extruded into the coagulation bath. The gravity of the polymer dope results in alignment of polymer chains, especially on the surface of the filament and increases the draw ratio of the filament [16]. However, electric networks between fillers can be disconnected by drawing, so the wet spinning process without drawing was adopted in this study.

In general, carbon nanomaterials such as carbon black (CB), carbon nanotubes (CNTs), graphene, and carbon nanofibers (CNFs) with a high aspect ratio have a large specific surface area several orders of magnitude higher than that of conventional fillers [17,18,19]. These are widely used to fabricate high-performance polymer nanocomposites due to their excellent mechanical properties and electrical conductivities [20]. In the case of CNTs, they are comprised of single or concentric-multiple walls of graphene or sp^2^-bonded carbon with a one-atom thickness. They are divided into single-, double-, and multi-walled according to the diameter and number of layers. Hence, the specific surface area varies accordingly. Strong attraction is induced between the CNTs due to the van der Waals forces, and agglomeration occurs. A smaller CNT diameter has a more highly curved and strained wall, making it less stable [7,17,20,21]. CNFs, also called vapor grown CNFs (VGCNFs), have larger diameters than single- and multi-wall CNTs, but a similar length and a single layer. They have a hollow core nanostructure composed of double layers of graphite planes stacked parallel to or at a specific angle from the fiber axis [18]. CNFs are much less expensive than CNTs, and are an excellent alternative to CNTs. In addition, the unique structure of the most common type of VGCNFs, which is cup-stacked, contains more reactive carbon edges that can be functionalized to interact with the matrix, thereby VGCNF dispersion, and enhances stress transfer from the polymer matrix to the nanofiller [19,21]. Polyacrylonitrile (PAN) is one of the most important polymers for preparing high-performance carbon fibers. Recently, carbon nanomaterial/PAN composite fibers with improved mechanical properties from the carbon fibers have been produced [8,9,10,11,12,13,14,15].

Previous studies have dealt with carbon nanomaterials for wearable device or sensors by using hydrogels, nanoparticles, nanocomposite, and nanofilms [22,23]. However, since the type of yarn is suitable for application to garments such as e-textiles or smart clothing, research on this is also being conducted. Previous studies manufacturing PAN-based carbon nanocomposite added fibers were conducted mainly to improve the mechanical strength by adding less than 1 wt% of carbon nanofillers [11,12,13,14,24,25,26]. To impart electrical properties, studies have been conducted using fillers with a high content of 15 wt% or more [8,9,10,11]. On the other hand, studies examining the rheological properties of composite solutions have been conducted to establish the appropriate spinning conditions because of the increased viscosity of the spinning dope. In addition, researchers have mentioned the possibility of wet-spinning processes, but there have been few studies on the electrical properties. Thus, studies on spinning conductive yarns by adding nano-scale particles such as carbon nanomaterials into a polymer are steadily progressing, but there is a limit to achieving high conductivity because an increase in filler content results in a deterioration of the physical properties of the material. Therefore, this study manufactured a polyacrylonitrile-based conductive yarn by wet spinning. Two types of carbon nanomaterials, CNT and CNF, were selected to prepare conductive yarns with varying contents of each material. After selecting the optimal dispersion method and viscosity by preparing a composite solution of two types of carbon nanomaterials alone or by mixing, a PAN-based conductive yarn was prepared through a wet spinning process. The morphology, mechanical properties, physical properties, and electrical properties of each sample were analyzed to select the optimal conditions.

## 2. Experimental

### 2.1. Materials

Polyacrylonitrile (PAN) was obtained from Taekwang Ind. Co. Ltd. (Seoul, Korea). The component of PAN was 87.1 wt% of acrylonitrile and 12.9 wt% vinyl acetate. Multi-wall carbon nanotubes (MWCNT, CM250, Hanhwa Nanotech, Incheon, Korea) and vapor-grown carbon nanofibers (VGCNF, PR-24-XT-HHT, Pyrograf Products, Inc., Cedarville, OH, USA) were used to produce the conductive fillers. Dimethyl sulfoxide (DMSO, 99.9%, SAMCHUN Chemical, Seoul, Korea) was used as a polymer solvent. Table 1 lists the specifications of the carbon nanomaterials in this study.

### 2.2. Preparation of a PAN-Based Conductive Composite Solution Using Carbon Nano-Material by the Dispersion Method

In this study, a composite solution was finally prepared using the following steps for three-stage wet spinning. First, this research team prepared a composite solution according to three dispersion methods by adding a conductive filler with a content of 16 wt% compared to the polymer. A first dispersion made of DMSO solvent blended with 16 wt% of CNT, 16 wt% of CNF, or a mixture of 8 wt% CNT and 8 wt% CNF corresponding to the polymer mass was prepared using different dispersion methods.

The first dispersion method was mechanical stirring (MSH-20D, Daihan Scientific Co. Ltd., Wonju, Korea). The material was dispersed for 24 h at 1000 rpm. The secondary method was ultrasonic machining (SONOPULS, BANDELIN Electronic GmbH & Co. KG, Berlin, Germany), it was dispersed three cycles for 65% amplitude sonication for 10 min. The last dispersion method was ball milling (Rotate ring mill, Armstec Ind. Co. Ltd., Hwaseong, Korea), dispersed for 4-h in three-steps by controlling each rotating axis speed and time. Subsequently, 10 wt% of PAN was added to the first dispersion to prepare a final composite solution through a co-rotation mixing system.

A conductive composite solution was prepared by selecting a final dispersion method suitable for wet spinning. The final composite solution was prepared by adding a high content of carbon nanomaterials corresponding to 16 wt%, 24 wt%, and 32 wt% of the polymer. A first dispersion was carried out in the same method as described above. Table 2 lists the composition of CNT, CNF, and CNT/CNF blend in the composite solution. Subsequently, 10 wt% of PAN was added to the first dispersion to prepare a final spinning dope using a co-rotation mixing system.

### 2.3. Preparation of PAN-Based Composite Film Based on the Wet Spinning Process

**Figure 1:**
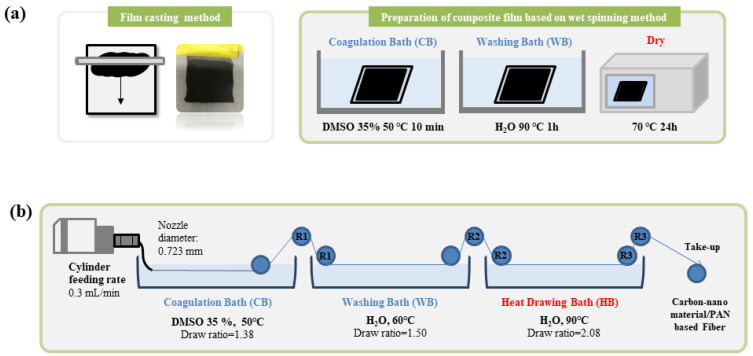
Schematic of the preparation process of (**a**) the PAN-based composite film and (**b**) wet spinning used in this study.

Before preparing the PAN-based conductive yarn, the composite films based on the wet-spinning process were manufactured. Figure 1a shows a scheme of the process of the composite film preparation of this study. The films were obtained using a solution casting method. A 1 mL sample of the composite solution was poured onto the glass substrate. The roller thickness was set to 10 μm, and the solution was applied uniformly on the glass substrate. Subsequently, the glass substrate coated with the composite solution was immersed into a DMSO/H_2_O coagulation bath (35/65 *w*/*w*) at 50 °C for 10 min. The composite film in the coagulation bath was washed in a water bath at 90 °C for one hour. The resulting composite films were finally dried in a 70 °C convection oven for 24 h.

### 2.4. Preparation of the PAN-Based Conductive Yarn Using a Three-Stage Wet Spinning Process

**Figure 2:**
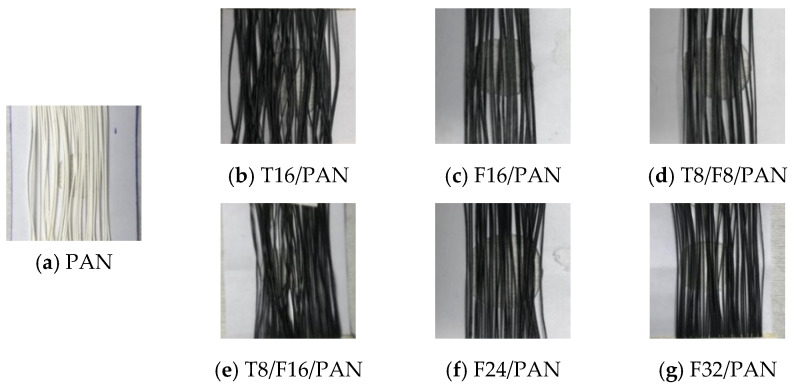
Obtained PAN-based conductive yarn through wet spinning. (**a**) PAN, (**b**) T16/PAN, (**c**) F16/PAN, (**d**) T8/F8/PAN, (**e**) T8/F16/PAN, (**f**) F24/PAN, (**g**) F32/PAN.

The PAN-based conductive yarns were prepared using a lab-scale three-stage wet spinning system. The detailed process is as follows. Figure 1b shows a schematic diagram of the lab-scale wet spinning condition and process. Among the three dispersion methods, the dispersion method finally selected for the production of a composite solution for the wet spinning process was ball-milling. The prepared spinning dope was spun into the DMSO/H_2_O coagulation bath (35/65 *w*/*w*) using a needle of 21 G with 0.723 mm diameter with a cylinder pump a cylinder feeding rate of 0.3 mL/min. At that time, the temperature of the spinning dope was maintained at 50 °C, and the draw ratio was 1.38. After the coagulation bath stage, the spun yarn was passed through a washing bath at 60 °C accompanied by a draw ratio of 1.50. The spun yarn was then passed through the heat drawing bath with a corresponding draw ratio of 2.08 at 90 °C. The resulting conductive yarns were finally dried in a 70 °C convection oven for 24 h. Figure 2 shows the finally obtained PAN-based conductive yarns.

### 2.5. Characterization

#### 2.5.1. Analysis of the PAN-Based Carbon Nanomaterial Added Composite Solution

- Viscosity

The properties of the PAN-based composite solution containing the carbon nanomaterials by a dispersion method and the contents of the conductive fillers, the viscosity of the PAN and PAN-based carbon nano-material composite solution was analyzed by a visual evaluation digital image and measured using a rotational rheometer with a small sample adapter on a Brookfield DV2T Viscometer (AMETEK Inc., Berwyn, IL, USA). The visual evaluation was conducted to confirm the change in the solution. A 0.5 g composite solution was applied to the glass substrate using a syringe, and a digital image was taken after 1 min. A test was conducted using a viscometer at 20 °C at 10 rpm three times to confirm this tendency quantitatively.

#### 2.5.2. Analysis of the PAN-Based Carbon Nanomaterial Added Composite Film

- Morphology

The cross-sectional morphology of a PAN-based conductive film, according to the contents and types of carbon nanomaterials, were analyzed by field emission scanning electron microscopy (FE-SEM, SU8010, Hitachi High-Technologies Corp., Tokyo, Japan). The images were taken at a magnification of ×200.

- Electrical property

Before manufacturing the PAN-based conductive yarn, to confirm the electrical properties according to the carbon nano-material content, the composite films based on the wet-spinning process were manufactured, and the resistance was measured. The electrical resistance of the PAN-based composite film containing the carbon nanomaterials with different contents was measured. The resistance per 1 cm was measured 10 times for three samples using a multi-meter (FLUKE 287, FLUKE Co. Ltd., Everett, WA, USA), and the mean value was used. Subsequently, the electrical resistance (R) was measured based on the AATCC-76 method. The sheet resistance (R_s_) was calculated using Equation (1):

R_s_(Ω/sq) = (W/D) × R (1)

where R is the resistance (Ω) measured using the multimeter; W is the width (mm) of the sample; and D (mm) is the distance between the two electrodes.

- Statistical analysis

*T*-test was used to numerically distinguish the differences of resistance in between the single and the mixture fillers, or the differences among the contents of the different fillers. The confidence level of the *t*-test was 95%. If the *p*-value was below 0.05, a comparison of two samples was considered as different. *T*-test results were derived by MATLAB R2021b.

#### 2.5.3. Analysis of the PAN-Based Carbon Nanomaterial Added Wet-Spinning Yarn

- Morphology

The cross-sectional morphology of PAN-based conductive yarn was examined by FE-SEM (SU8010, Hitachi High-Technologies Corp., Tokyo, Japan). For the cross-section morphology, the fibers were immersed in liquid nitrogen and fractured carefully. The images were taken at magnifications of ×200 and ×20,000.

- Wide-angle X-ray diffraction and crystal orientation property

The 2D wide-angle X-ray diffraction (WAXD, D8 Discovery, Bruker, Billerica, Germany) of the PAN-based conductive yarn was carried out to measure the 2-theta angle and crystal orientation of the wet-spun fiber. WAXD was performed using CuKα radiation (λ = 0.1548 nm) at a voltage and current of 50 kV and 1000 μA, respectively. The 360° azimuthal circle was used to permit the fiber axis to rotate 360° about the vertical. The orientation of the PAN-based conductive yarn used by carbon nanomaterials was calculated using Equation (2):

(2) $$\mathrm{Crystal}\;\mathrm{orientation}=\frac{360-FWMH}{360}\times 100\;\left(\%\right)$$

where *FWMH* is the half-value width in degrees of the curve of intensity against the azimuthal angle.

In addition, the crystallinity of the fiber was calculated using Equation (3) [29]:

(3) $$\mathrm{Crystallinity}=\frac{Peak\;area}{Total\;area}\times 100\;\left(\%\right)$$

- Mechanical property

To examine the mechanical properties of a single fiber, the tenacity, elongation, and fineness were measured using a Favimat fiber test system (Favimat-airobot2, Textechno H. Stein GmbH & Co. KG, Monchengladbach, Germany). The tensile test of fibers was carried out 10 times at a crosshead speed of 2 mm/min and a gauge length of 20 mm.

- Thermal property

The thermal properties of the PAN-based conductive yarn used by carbon nanomaterials were determined by thermogravimetric analysis (TGA, Q500, TA Instruments Inc., New Castle, DE, USA). TGA was investigated in nitrogen gas at a heating rate of 20 °C/min. The measurement temperature ranged from 30 °C to 800 °C. The TGA curve and derivatives curves were obtained, and the temperature at transition (T_trans_) and maximum rate (T_mr_), and the residue at temperature at the maximum rate and 750 °C were analyzed.

- Electrical property

The electrical properties of the PAN-based conductive yarn used by the carbon nanomaterials were confirmed by measuring the resistivity using a multi-meter (FLUKE 287, FLUKE Co. Ltd., Everett, WA, USA). The resistivity per cm of the samples was examined 10 times for three samples, and the mean value was used.

- Statistical analysis

The resistance difference of conductive yarn samples was analyzed in the same *t*-test calculation and confidence level as conducted in the composite films. 

## 3. Results and Discussion

### 3.1. Viscosity of PAN-Based Composite Solution with Carbon-Nano Materials

#### 3.1.1. By Different Dispersion Method

**Figure 3:**
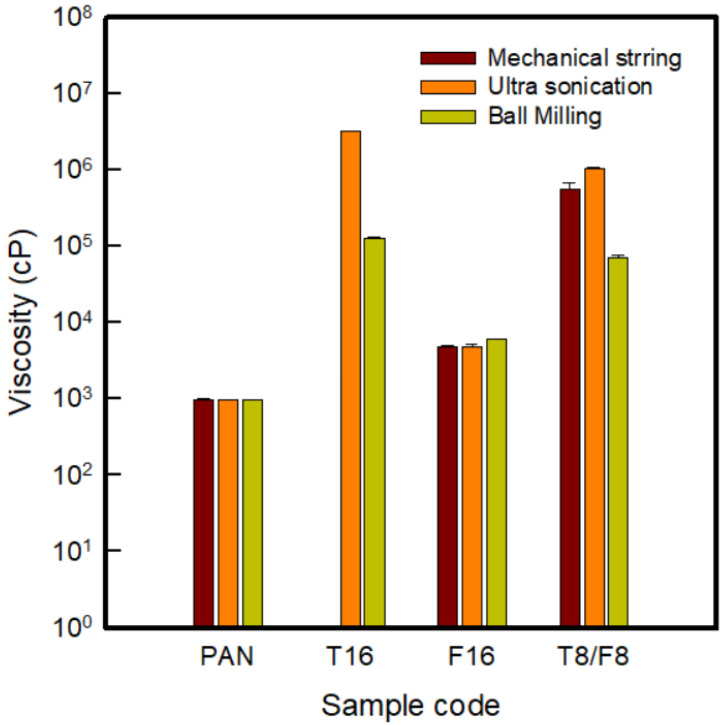
Viscosity of the PAN-based composite solution corresponding to the content of 16 wt% carbon nanomaterials by different dispersion methods.

Figure 3 shows the viscosity of the secondary dispersion. Approximately 16 wt% of the carbon nanomaterial was added to the polymer content to compare the viscosity of the composite solution according to the type of carbon nanomaterial and the dispersion method. In this study, when preparing the primary dispersion using carbon nanomaterials and DMSO, the dispersion method was different with mechanical stirring, ultra-sonication, and ball milling.

Figure 3 indicates the viscosity of the PAN-based composite solution corresponding to the content of 16 wt% carbon nanomaterials by different dispersion methods. As a result of the digital image analysis in Figure S1, it was shown that the three types of composite solutions after ball milling were better spread over the glass substrate than with the other two methods. Measurements of the viscosity to confirm quantitatively, in the case of pure PAN, revealed a value of approximately 960 cP, showing that the viscosity increased after adding the carbon nanomaterial. When analyzed by carbon nano-material type, the viscosity tended to increase in the order of F16/PAN < T8/F8/PAN < T16/PAN, which can be explained by the influence of the structures of MWCNTs and VGCNFs [7,18,19,21]. Carbon nanotubes, in which the specific surface area varies depending on the diameter and number of layers, induces a strong attraction, particularly due to van der Waals forces, causing aggregation [7,21]. VGCNFs are hollow nanofibers with a lower viscosity because the dispersibility is easier than that of CNTs. When CNTs and CNFs were incorporated together, the viscosity of T8/F8/PAN appeared between T16/PAN and F16/PAN because of their structural characteristics.

Through the dispersion methods, the composite solution with each carbon nanomaterial showed different viscosity values. T16 stirred with mechanical stirring showed a high viscosity exceeding the measurable viscosity range, and T16 dispersed through ultra-sonication and ball milling showed viscosity values of 2,600,000 ± 99,000 cP and 420,000 ± 2400 cP, respectively. As a result, the viscosity decreased after ball milling. As mentioned above, MWCNTs aggregate easily to form bundles or ropes, so preparing a well-dispersed dope for wet spinning is the most important factor. Accordingly, in this study, to reduce the viscosity by increasing the dispersibility of the wet spinning dope containing MWCNTs, which has the highest viscosity, the ball milling process was confirmed to be more effective than conventional mechanical stirring and ultra-sonication. On the other hand, in the case of F16/PAN, similar viscosities were exhibited in the range of approximately 1800–2400 cP with the three dispersion methods. This was attributed to the relatively good dispersion without aggregation because of the structural difference between MWCNTs and VGCNFs. Furthermore, the viscosity of T8/F8/PAN, in which two different materials were blended, was affected by the dispersion of the MWCNTs, and decreased approximately 40 times from approximately 770,000 cP to 18,000 cP after ball milling when compared to the other dispersion methods. Finally, this study selected ball milling to prepare a dope for wet spinning.

#### 3.1.2. By Different Contents of Carbon Nanomaterials

**Figure 4:**
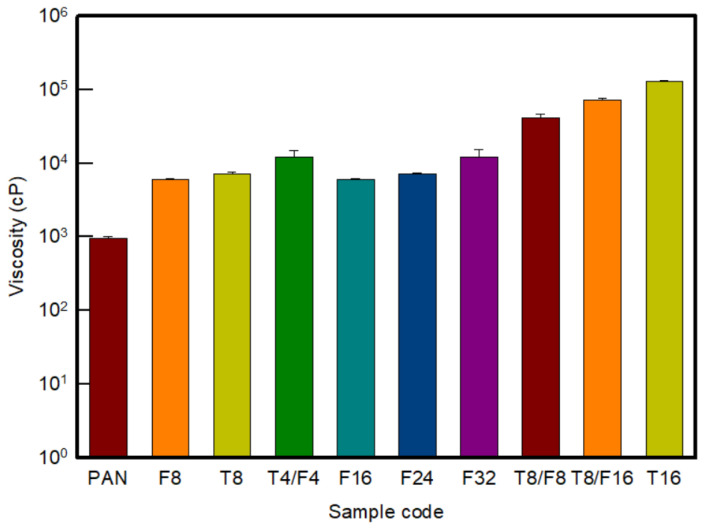
Viscosity of the PAN-based composite solution with various contents of carbon nanomaterials.

Figure 4 and Figure S2 shows the viscosity and digital images according to the type and content of carbon nanomaterials manufactured based on the previously selected ball milling dispersion method in the graph. In this study, based on the CNT [8,9] and CNF [10] contents of previous studies that performed wet spinning based on carbon nanomaterials, the content of each carbon nanomaterial was adjusted to 8 wt%, 16 wt%, 24 wt%, and 32 wt% was selected to prepare a composite solution, and the viscosity of the prepared solution was confirmed.

As seen in the results, the viscosity increased in the order of CNF/PAN < CNT/CNF/PAN < CNT/PAN as in the above result. The viscosity of T16/PAN was 10^5^ cP, which was higher than that of F32/PAN or T8/F16/PAN. Therefore, through the ball milling process selected in this study, it was possible to prepare a solution exhibiting a viscosity ranging from 10^3^ cP to 10^5^ cP in general, despite the addition of a high content of carbon nanomaterials.

### 3.2. Electrical Property of PAN-Based Composite Film with Different Contents of Carbon-Nanomaterials

**Figure 5:**
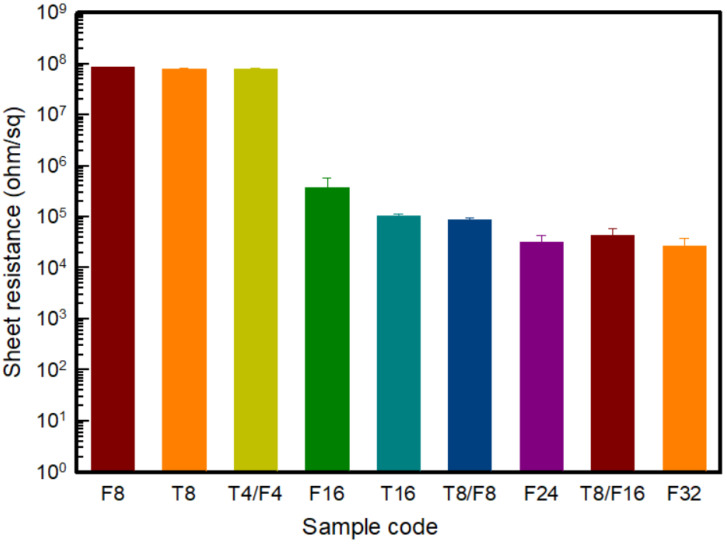
Sheet resistance of the PAN-based composite films with various carbon nanomaterial contents.

Figure 5 shows the sheet resistance by manufacturing a PAN-based composite film with different contents of carbon nanomaterials to confirm the electrical properties according to the type and content of carbon nanomaterials before manufacturing the conductive yarn. In general, the resistance range of conductive materials is less than 10^6^ Ω/sq [18]. Experimentally, resistance values exceeding 10^6^ Ω/sq were obtained in the case of Pure PAN without a conductive filler and T8/PAN, F8/PAN, and T4/F4/PAN with carbon nanomaterials with an 8 wt% content. Hence, pure PAN cannot be used as a conductive material. On the other hand, when the carbon nanomaterial content was added at 16 wt% or more, the sheet resistance was less than 10^6^ Ω/sq, indicating the range of the conductive materials.

In the case of incorporating a single material into PAN, the resistance of F16/PAN and T16/PAN were 380 ± 190 kΩ/sq and 103 ± 100 kΩ/sq, respectively, the point at which the carbon nanomaterials formed a conductive path. Accordingly, the sheet resistance of F16/PAN, F24/PAN, and F32/PAN, according to the increase in CNF content, was confirmed to be 380 ± 190 kΩ/sq, 45 ± 15 kΩ/sq, and 27 ± 11 kΩ/sq, respectively, showing a tendency to decrease. The resistance decreased because a conductive network formed from the addition of more CNF than the content required to form a conductive path in the PAN polymer [18]. In addition, the sheet resistance of T16/PAN was approximately three times lower than that of F16/PAN when the same amount of carbon nanomaterials was added. In the case of conductive fillers, the conductivity was reduced rather than having good distribution and dispersion in the polymer [7,18]. Accordingly, unlike CNF, which has good distribution and dispersion in the polymer because of its easy dispersion and low cohesive force, CNTs have good dispersion by ball milling in the polymer, but has a poor distribution because of the cohesive force, thereby forming more conductive paths. Hence, sheet resistance tended to decrease.

The sheet resistance of the CNT and CNF mixed material was examined, and the sheet resistances were compared through the *t*-Test. In Table 3, pairs of T8/F8 to either T16 or F16 showed low *p*-value under 0.05. The sheet resistance of T8/F8/PAN was significantly lower than that of T16/PAN and F16/PAN. In addition, in the case of T8/F16/PAN, the sheet resistance was 32 ± 12 kΩ/sq, which was lower than that of a single F24/PAN, and similar to F32/PAN. This means that the mixture of CNTs and CNFs can improve electric conductivity rather than single filler composites. On the other hand, it was observed that there was no difference in the comparison of T8/F16 to F24, and F24 to F32. In the previous study of incorporating the different types of carbon nanomaterials, a synergistic effect was observed after mixing carbon-based fillers of different sizes and structures [20]. In the case of CNTs and CNFs, they had a one-dimensional rod-like shape and similar diameters and lengths. On the other hand, when two materials with different specific surface areas were mixed, the content of CNTs with high aggregation in the polymer matrix was reduced, and CNFs with excellent dispersibility were added. Accordingly, the sheet resistance decreased because the contact area that can form electrical percolation was improved. Thus, using T8/F16/PAN, a film with excellent electrical properties can be prepared with a low-viscosity solution.

### 3.3. Morphology of the PAN-Based Composite Film and Yarn with Different Contents of Carbon-Nano Materials

Table 4 and Table 5 present carbon nanomaterials and cross-section images of each sample to confirm the morphology according to the type and content of carbon nanomaterials. Here, the results of more than 16 wt% carbon nanomaterials/PAN samples, in which the electrical properties were confirmed through the composite film results, are presented.

The CNTs existed in the form of bundles because of the strong attraction caused by van der Waals forces. In the case of CNF, however, there was no aggregated region. 

The films and yarns according to the content of carbon nanomaterials suggest that the cross-section area tends to fill with increasing contents of CNTs or CNFs. In addition, a PAN-based conductive yarn containing a high content of 16 wt% to 32 wt% can be manufactured continuously through a three-step wet spinning process. An analysis of the morphology of each sample showed that, in the pure PAN film, the presence of voids was observed inside, which was also confirmed in the yarn. In particular, in the case of yarn, the outer and inner areas were distinguished, and the inner area was relatively filled compared to the outer area. This is because the outer area is exposed more easily than the inner area during the coagulation process. When compared according to the carbon nano-material type, the T16/PAN film appears to be the most filled in the case of the film, but it was rougher than the other samples. On the other hand, in F16/PAN, F24/PAN, and F32/PAN, voids were observed rather than on the T16/PAN film, but the morphology was smoother. The mixed T8/F8/PAN and T8/F16/PAN showed fewer voids according to the content, and they were smoother than T16/PAN. Hence, they showed a similar trend to the PAN-based conductive yarn. Additionally, the CNTs exist in the form of bundles due to the strong attraction.

To present the images in Table 5 for more detailed observations, Table 6 presents the PAN-based conductive yarn with different carbon nanomaterial contents at ×20,000 magnifications. Compared to the pure PAN fiber, as carbon nanomaterials were added, the portion containing the polymer decreased, and the portion of the conductive filler increased. Accordingly, the number of adjacent conductive fillers increased with increasing content. Hence, a conductive path can form an electrical percolation threshold because of these morphological characteristics.

### 3.4. Wide-Angle X-Ray Diffraction (WAXD) and Crystal Orientation of PAN-Based Conductive Yarn with Different Contents of Carbon-Nano Materials

**Figure 6:**
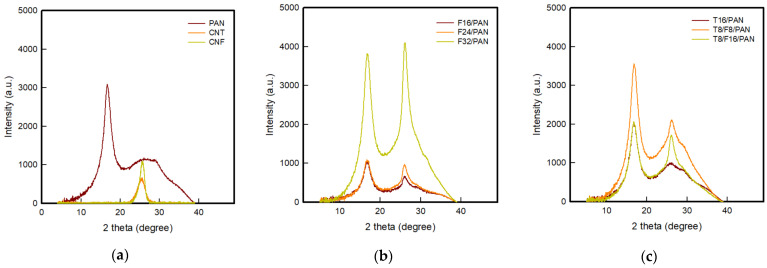
XRD pattern of the PAN-based conductive yarn with various contents of carbon nanomaterials (**a**) Raw materials, (**b**) CNF, and (**c**) CNT and CNT/CNF blended.

**Figure 7:**
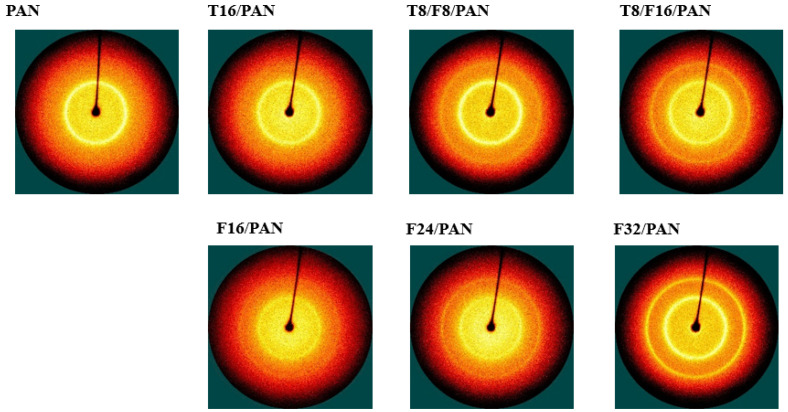
2D WAXD patterns of the PAN-based conductive yarn by different contents of carbon nanomaterials.

Figure 6 and Figure 7 present the XRD patterns and 2D WAXD patterns of the PAN-based conductive yarn according to the type and content of carbon nanomaterials. Using these results, the percentage of crystalline, amorphous, and crystal orientation of samples were confirmed, as shown in Table 5. The strong peak of the pure PAN fiber appeared at approximately 17° and 29.5° 2θ, which was attributed to the crystal peak of the (200) and (020) planes, and the broad peak at approximately 25° 2θ corresponded to the amorphous peak [12,25]. The crystalline (%) and amorphous (%) percentages were 28.4% and 71.6%, respectively, confirming the higher amorphous content than crystalline content. For the CNTs and CNFs, a narrow and intense peak at 26.5° 2θ was noted, which corresponded to the (002) plane of CNTs and CNFs [25]. Accordingly, in the PAN-based yarn to which the carbon nanomaterials were added, the peak intensity near 26.5° 2θ increased with increasing carbon nanomaterial content, with a concomitant decrease in the intensity of the 29.5° 2θ peak of PAN. The PAN peak size obtained from the (200) XRD peak in the T/PAN or F/PAN or T/F/PAN sample was larger than that in the pure PAN sample. The d-spacing of the (200) plane of PAN decreased with increasing carbon nanomaterial content, indicating a closer packing of PAN molecules in the crystal [25]. Thus, the crystal structure of the PAN fiber was improved after mixing CNT, CNF, and CNT/CNF, as listed in Table 5 through the crystallinity (%). The crystallinity (%) increased in the following order: CNT < CNF < CNT/CNF. Moreover, the crystallinity increased with increasing carbon nanomaterial contents. The amorphous region of the PAN polymer decreased as the carbon nanomaterial with a crystal structure was added. In addition, the crystallinity was improved after mixing, after adding the fibers of the carbon nanomaterials either singly or mixed with the same content. As described above, as two materials with different specific surface areas are mixed, the content of CNTs with high aggregation in the polymer matrix was reduced, and packing was further improved by adding CNFs with excellent dispersibility.

The crystal orientation (%) was measured based on azimuthal angle analysis, and the results are listed in Table 7. Different results were shown for each carbon nanomaterial. The CNT-added samples for T16/PAN, T8/F8/PAN, and T8/F16/PAN showed 70.3 %, 74.1 %, and 71.7 % lower values than the PAN fiber, respectively. On the other hand, the CNF-added F16/PAN, F24/PAN and F32/PAN showed 81.8%, 80.8%, and 77.2% higher values than the PAN fiber, respectively. Unlike CNTs, which use the bundle form due to aggregation, CNFs with excellent dispersibility in the polymer were added to form a rheological percolation threshold. The crystal orientation increased as the stretching process performed during wet spinning was added [7]. In addition, crystal orientation tended to decrease with the increasing content of conductive fillers in single/mixed carbon nanomaterials. The draw for PAN/carbon nanomaterial composite fibers decreased with an increased content of carbon nanomaterials when wet spinning under the same conditions [11]. Therefore, wet spinning-based fibers with the appropriate crystalline orientation can be prepared when the two materials are mixed because increasing the content of carbon nanomaterials increases the crystallinity, but decreases the orientation.

### 3.5. Mechanical Properties of PAN-Based Conductive Yarn with Different Contents of Carbon Nanomaterials

**Figure 8:**
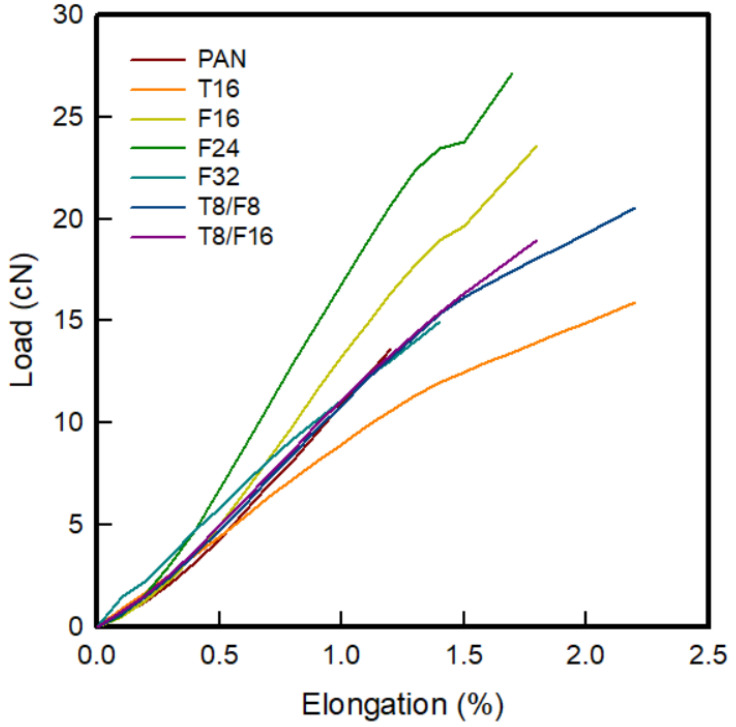
Load–elongation curve of the PAN-based composite yarn with different contents of carbon nanomaterials.

Figure 8 presents the load–elongation curve of PAN-based conductive yarn with different contents of carbon nanomaterials. According to Figure 8, Table 8 lists the single fiber tensile test results of the PAN-based fibers according to the type and content of carbon nanomaterials. The tenacity of the pure PAN fiber was 2.46 ± 0.50 g/den. In the case of T16/PAN, T8/F8/PAN, and T8/F16/PAN to which CNTs had been added, the values were 1.24 ± 0.17, 2.18 ± 0.75, and 1.97 ± 0.30 g/den, respectively, which were lower than those of the PAN fiber. The tenacity of F16/PAN, F24/PAN, and F32/PAN containing CNF was 3.44 ± 1.04, 3.17 ± 0.78, and 0.60 ± 0.11 g/den. The two samples, except for F32/PAN, showed higher tenacity than the PAN fiber, but decreased with increasing content, and fracture occurred at less than 2.5% elongation. In general, when a small quantity of carbon nanofillers (<1 wt%) was added, the mechanical strength can be improved because it acts as a linkage connecting the crystal and the amorphous regions [11,12,13]. On the other hand, the tensile properties may deteriorate when a high content of carbon nanomaterials is added to the PAN polymer because of two effects. The first is the “side effect”, in which phase separation occurs due to the heterogeneous distribution of carbon nanomaterials and polymers caused by aggregation as the contents of the carbon nanomaterials increase. The second can be explained by the “end effect” caused by voids that occur because the polymer cannot fill the end of the carbon nanomaterial [10,14]. Nevertheless, in this study, by selecting a suitable dispersion method for wet spinning, a PAN-based conductive yarn with similar or improved mechanical properties to CNF of F24 or less or pure PAN when mixed was possible.

### 3.6. Thermal Property of the PAN-Based Conductive Yarn with Different Contents of Carbon Nanomaterials

**Figure 9:**
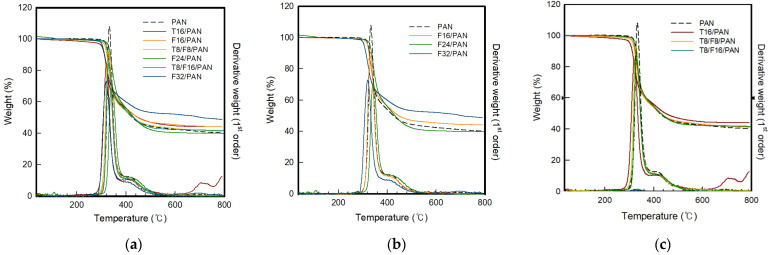
TGA and first derivative curve of PAN-based conductive yarn with various contents of carbon nanomaterials (**a**) Total, (**b**) CNF, and (**c**) blended CNT and CNT/CNF.

The thermal stability of PAN-based conductive yarn used by carbon nanomaterials was investigated using TGA under a nitrogen environment. Figure 9 shows the weight loss and first derivative curves of the samples, Table 9 lists the TGA data. Pure PAN and conductive yarn containing carbon nanomaterials showed a sharp weight loss between 300 and 500 °C. The T_trans_ and T_mr_ range was approximately 320 to 330 °C, and approximately 370 to 390 °C, respectively. Generally, the PAN polymer can be converted to a cyclic ladder structure in the temperature range of 200–350 °C, even in an inert environment [10]. This cyclic structure can be carbonized upon further heating under an inert environment, leading to an amorphous carbonaceous structure (char).

Pure PAN showed a T_trans_ and T_mr_ of 332.8 °C and 389.5 °C, respectively, from TGA. The T_trans_ and T_mr_ decreased as the weight percentage of carbon nanofillers was increased. The decrease in the degradation temperature is related to the increased thermal conductivity imparted by the added nano-fillers, which have been shown in many studies [30,31,32]. In addition, Figure 9a–c shows that T_trans_ varies according to the type of carbon nanomaterial. In the case of F16/PAN and F24/PAN spun by mixing CNF, the transition temperature was higher than that of T16/PAN, T8/F8/PAN, and T8/F16/PAN spun by mixing CNT. This was attributed to the shape of the CNT and CNF. In the case of CNF used in this study, T_trans_ appeared to be delayed due to the layered graphite structure having a fully graphitized form [14]. Accordingly, the transition temperature of T8/F8/PAN and T8/F16/PAN spun by mixing CNT and CNF was delayed compared to T16/PAN. In the case of char residue, 40%–50% residue was observed at 750 °C overall. At this time, the residue at 750 °C showed the lowest value of 40.1% for PAN, and tended to increase slightly with increasing carbon nanomaterial content. This was attributed to the CNT or CNF content because the PAN polymer content decreased with increasing carbon nano-material content. Therefore, the two carbon nanomaterials with excellent thermal properties influenced the char residue.

### 3.7. Electrical Property of the PAN-Based Composite Yarn with Different Contents of Carbon Nano Materials

**Figure 10:**
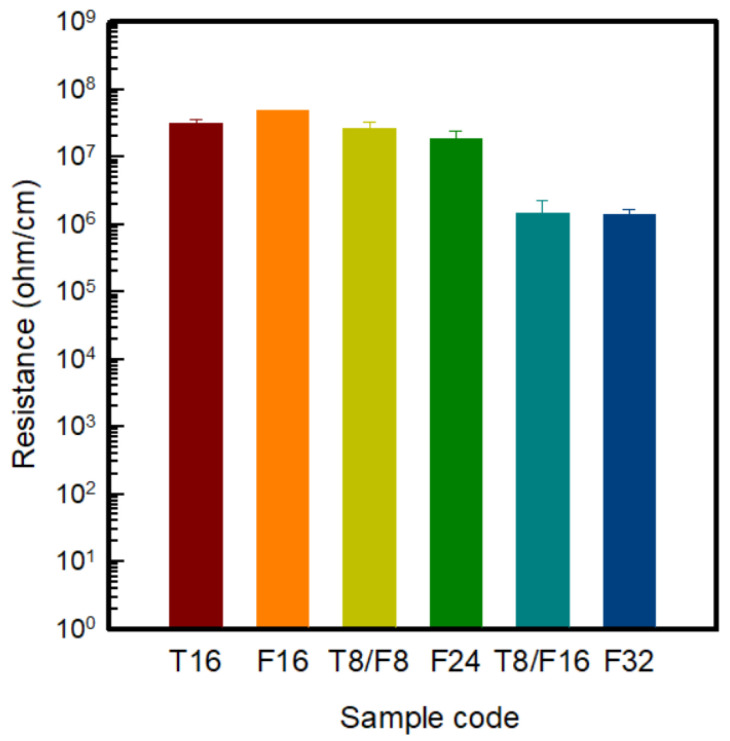
Resistance of the PAN-based conductive yarn with various carbon nanomaterial contents.

Figure 10 shows the resistance of PAN-based conductive yarns according to the type and content of carbon nanomaterials manufactured based on wet spinning. The overall trend was similar to that observed in the composite film, but ten times decreased in all samples when the resistance was measured. The result was based on the formula in which the resistance is proportional to the length and inversely proportional to the cross-sectional area. In a previous study analyzing the resistance of MWCNT/polymer composites with injection and compression molds of different thicknesses, the resistance was lower than that of the injection mold when the compression mold had a large area. Moreover, the thickness was lower than that of the thin film [16]. In this study, the film had a large area, while the fiber had a long and thin shape, so the resistance appeared to have increased. Accordingly, 16 wt% and 24 wt% of the carbon nanomaterial-based PAN-based yarn showed a relatively high resistance of 10^7^ Ω/sq. On the other hand, when F32/PAN was added, it reached a range representing a conductive material at 10^6^ Ω/sq. At this time, in the case of T8/F16/PAN, the resistance value was similar to that of F32/PAN. As seen in Table 10, the T8/F16/PAN sample and F32/PAN sample had 0.887 *p*-value so that the resistance of those two samples were not significantly different. In other words, the mixture of T8/F16 can achieve a resistance as low as F32. On the other hand, F24/PAN and F16/T8/PAN showed a 0.00001 *p*-value so that resistances of F16/T8 and F24 were significantly different. F8/T8 also had significantly less resistance than F16. This means that a mixture of CNT and CNF can decrease resistance less than that of the same weight percentage of a single filler. As above-mentioned, as two materials with different specific surface areas are mixed, the content of CNTs with high aggregation in the polymer matrix is reduced. In addition, the contact area that can form electrical percolation is improved by adding CNF with excellent dispersibility. Therefore, after mixing CNF and CNF, the electrical properties were improved, and the yarn could be used as a conductive material for T8/F16/PAN.

## 4. Conclusions

This study manufactured a polyacrylonitrile-based conductive yarn by wet spinning. Two types of carbon nanomaterials were selected to produce conductive yarns with varying contents of each material, and then properties of conductive yarn were analyzed.

To control the optimal dispersion and solution conditions for the wet spinning process, a PAN-based composite solution using two types of carbon nanomaterials suitable for the wet spinning process were prepared. The samples were characterized according to three dispersion methods: mechanical stirring, ultra-sonication, and ball milling. The viscosity of each solution was measured, and it was confirmed that CNT/PAN, CNF/PAN, and CNT/CNF/PAN composite solutions had the lowest viscosity after using the ball milling method. Additionally, it was confirmed that the composite solution prepared by ball milling exhibited a viscosity within a similar range even when the content of carbon nanomaterials increased. Accordingly, the ball milling was adopted for wet spinning. To confirm the conditions under which the electrical characteristics appear, a PAN-based composite solution was prepared by selecting a high content of carbon nanomaterials of 8 wt%, 16 wt%, 24 wt%, and 32 wt% to control the viscosity. Based on the prepared wet-spinning solution, a film was prepared based on the wet spinning process and confirmed by measuring the surface resistance. It was less than 10^6^ Ω/sq at 16 wt% or more, indicating conductivity. According to the results, for wet-spinning, a PAN-based composite solution with a carbon nanomaterial content of 16 wt% or more was selected. 

To fabricate the conductive yarn, a PAN-based conductive yarn using a carbon nanomaterial with a high content from 16 wt% to 32 wt% was manufactured and its characteristics were analyzed. As the carbon nanofiller content increased, the conductive path improved as the distance between the carbon nanofillers decreased. WAXD showed that the crystallinity was improved after mixing the different carbon nanomaterials. The strength and tenacity increased in the order of CNT/PAN < CNT/CNF/PAN < CNF/PAN < PAN, and a PAN-based conductive yarn with similar or improved mechanical properties to that of pure PAN when mixed or CNF below F24 could be produced by selecting a dispersion method suitable for wet spinning. Finally, F24/PAN and T8/F16/PAN, had a similar electrical resistance to that of F32/PAN, and it was concluded that the 24 wt% of carbon nanomaterial contents achieved a decent resistance over the percolation threshold.

The electrical properties improved when two-types of carbon nanomaterials were mixed compared to those used alone at the same mass. The physical properties were also improved compared to when only CNTs were added. Thus, this material is expected to be applicable to various smart textiles and wearable devices because of its improved physical properties such as strength and conductivity through further research.

## Figures and Tables

**Table 1:** Specification of carbon nanomaterials in this study [27,28].

Types of Carbon Nanomaterials	Parameters				
	Diameter (nm)	Length (µm)	Surface Area (m^2^/g)	Molecular Weight (g/mol)	Purity Rating (wt%)
MWCNT [27]	50–200	50–200	41	12	>95
CNF [28]	60–100	≤200	225	12	>98

**Table 2:** Composition of CNF and CNT for the conductive composite solution.

Type	Code	Volume of Carbon Nano Fillers (g)					
PAN	PAN	10					
MWCNT	T	1.6	0.0	0.8	0.8	0.0	0.0
CNF	F	0.0	1.6	0.8	1.6	2.4	3.2
Total content of CNF and CNT (wt%)		16	16	16	24	24	32

**Table 3:** Results of the paired *t*-test analysis of PAN-based composite films with various types of carbon nanomaterials and contents.

Pair of *t*-Test	Mean (kΩ/sq)	S.D. (kΩ/sq)	*p*-Value
F16	380.0	19.0	0.001
T8/F8	86.0	9.9	
T16	103.0	11.0	0.003
T8/F8	86.0	9.9	
F16	380.0	19.0	0.002
T16	103.0	11.0	
T8/F16	44.0	12.0	0.074
F24	32.0	12.0	
T8/F16	44.0	12.0	0.010
F32	26.0	11.0	
F24	32.0	12.0	0.277
F32	26.0	11.0	

**Table 4:** Morphology of the types of carbon nanomaterials.

Type	CNT		CNF	
**Magnification**	**×200**	**×20,000**	**×200**	**×20,000**
**Image**	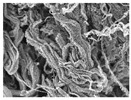	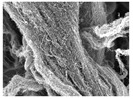	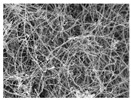	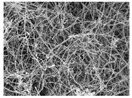

**Table 5:** Morphology of the PAN-based composite film and yarn with different contents of carbon nanomaterials (×200).

Contents of Carbon Nanomaterials in PAN-Based Composite (wt%)						
**PAN**	T16	F16	F24	F32	T8/F8	T8/F16
** 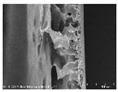 **	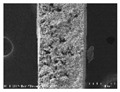	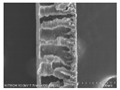	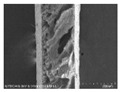	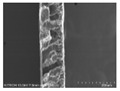	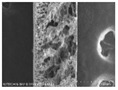	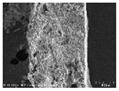
** 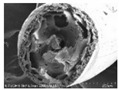 **	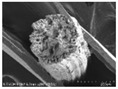	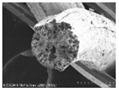	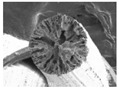	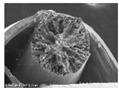	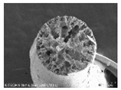	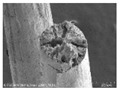

**Table 6:** Morphology of the PAN-based conductive yarn with different contents of carbon nanomaterials (×20,000).

Contents of Carbon Nanomaterials in PAN-Based Composite (wt%)						
PAN	T16	F16	F24	F32	T8/F8	T8/F16
** 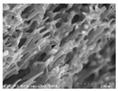 **	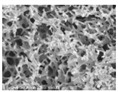	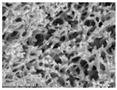	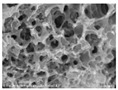	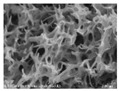	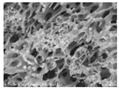	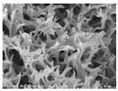

**Table 7:** Degree of the crystal orientation in the PAN-based composite yarn with different contents of carbon nanomaterials.

Parameter	Contents of Carbon Nanomaterials in PAN Based Composite (wt%)						
	PAN	T16	F16	F24	F32	T8/F8	T8/F16
Crystalline region (%)	28.4	30.0	32.0	34.6	39.6	33.6	35.3
Amorphous region (%)	71.6	70.0	68.0	65.4	60.4	66.4	64.7
Crystal Orientation (%)	74.9	70.3	81.8	80.8	77.2	74.1	71.7

**Table 8:** Mechanical properties of the PAN-based composite yarn with different contents of carbon nanomaterials.

Sample Code	Tenacity (g/den)	Elongation (%)	Linear Density (den)
PAN	2.46 ± 0.50	1.33 ± 0.22	7.42 ± 0.67
T16	1.24 ± 0.17	2.38 ± 0.64	15.87 ± 1.04
F16	3.44 ± 1.04	1.98 ± 0.36	8.35 ± 0.90
F24	3.17 ± 0.78	1.69 ± 0.30	9.64 ± 2.21
F32	0.60 ± 0.11	1.14 ± 0.21	25.85 ± 5.23
T8/F8	2.18 ± 0.75	2.33 ± 0.75	11.95 ± 0.50
T8/F16	1.97 ± 0.30	1.90 ± 0.33	13.15 ± 0.92

**Table 9:** TGA of the PAN-based composite yarn with different contents of carbon nanomaterials.

Sample Code	* T_trans_ (°C)	** T_mr_ (°C)	Residue at T_mr_ (%)	Residue at 750 °C (%)
PAN	332.8	389.5	55.7	40.1
T16	321.3	380.1	58.3	44.1
F16	330.9	380.6	57.6	42.2
F24	331.7	384.4	59.5	44.2
F32	320.6	374.8	62.8	48.6
T8/F8	328.0	375.0	57.6	41.1
T8/F16	330.2	376.7	64.2	41.8

* T_trans_: Transition Temperature/** T_mr_: Temperature at the maximum rate.

**Table 10:** Results of the paired *t*-test analysis of PAN-based composite fibers with various types of carbon nanomaterials and contents.

Pair of *t*-Test	Mean (MΩ/sq)	S.D. (MΩ/sq)	*p*-Value
F16	50.0	0.0	0.000
T8/F8	26.0	6.0	
T16	31.0	4.0	0.080
T8/F8	26.0	6.0	
F16	31.0	4.0	0.000
T16	50.0	0.0	
T8/F16	1.4	0.8	0.011
F24	18.0	6.0	
T8/F16	1.4	0.8	0.887
F32	1.4	0.3	
F24	18.0	6.0	0.000
F32	1.4	0.3	

## Data Availability

The data presented in this study are available in this article.

## Supplementary Materials

The following are available online at https://www.mdpi.com/article/10.3390/polym13203544/s1, Figure S1: Digital image of the PAN-based composite solution corresponding to the content of 16 wt% carbon nanomaterials by different dispersion methods, Figure S2: Visualized image of the PAN-based composite solution with various contents of carbon nanomaterials.
