# Supplementary material for: Process Optimization for Manufacturing PAN-Based Conductive Yarn with Carbon Nanomaterials through Wet Spinning
Source: Polymers (Basel). 2021 Oct 14;13(20):3544. doi: 10.3390/polym13203544 (PMC8537922; doi:10.3390/polym13203544)
Supplement: Supplementary file 1 [file polymers-13-03544-s001.zip › polymers-1380133-supplementary.pdf]

Supplementary Information

Process Optimization for Manufacturing PAN-based Conductive Yarn with Carbon Nanomaterials through Wet Spinning

Hyelim Kim[1], Hyeongmin Moon[1], Daeyoung Lim[1] and Wonyoung Jeong[1,*]

[1]Material and Component Convergence R&D Department, KITECH, Ansan, Gyeonggi-do, 15588, South Korea; hyelim1221@kitech.re.kr (H. K.); hm9487@kitech.re.kr (H. M.); zoro1967@kitech.re.kr (D. L.), wyjeong@kitech.re.kr (W. J.)

*Correspondence: wyjeong@kitech.re.kr

To confirm the visual evaluation of viscosity of composite solution, a 0.5 g composite solution was applied to the glass substrate using a syringe, and a digital image was taken after 1 min. As a result of digital image analysis in Figure S1, T16/PAN maintained its shape because of its higher viscosity than F16/PAN and T8/F8/PAN when dispersed using the three dispersion methods. The F16/PAN composite solution spread over the glass substrate because of its low viscosity after one minute, regardless of the dispersion method. In the case of the mixture of two types of carbon nanomaterials, the viscosity was intermediate between T16/PAN and F16/PAN. In addition, in terms of the dispersion methods, the three types of composite solutions after ball milling were better spread over the glass substrate than with the other two methods.

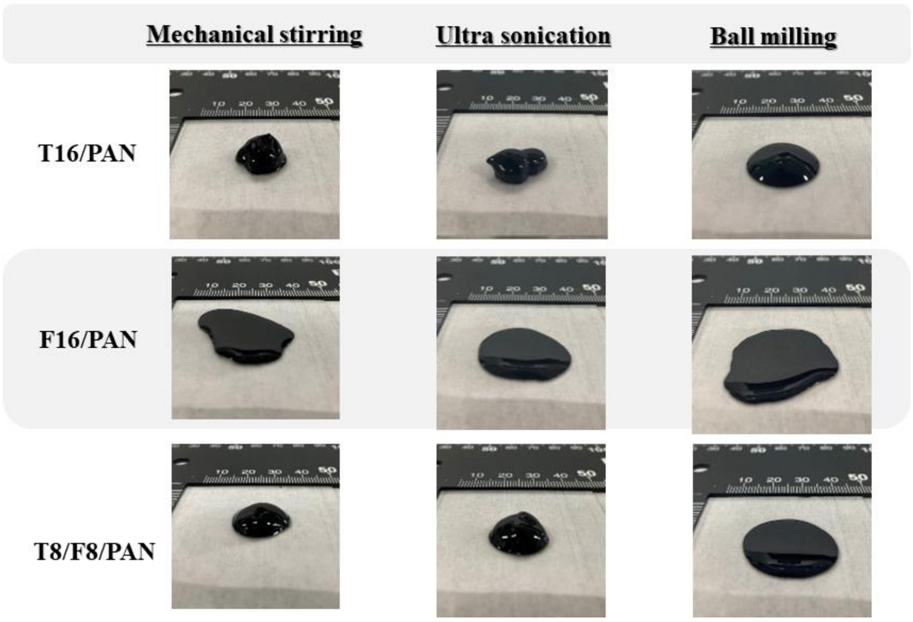

Figure S1. Digital images of the PAN-based composite solution corresponding to the content of 16 wt% carbon nanomaterials by different dispersion methods.

As shown in Figure S2, when the viscosity of the secondary dispersion after ball milling dispersion was confirmed visually through a digital image, the surface tension of T16/PAN appeared to be highest, and other composite solutions were similar to Pure PAN, which is spread over the glass substrate.

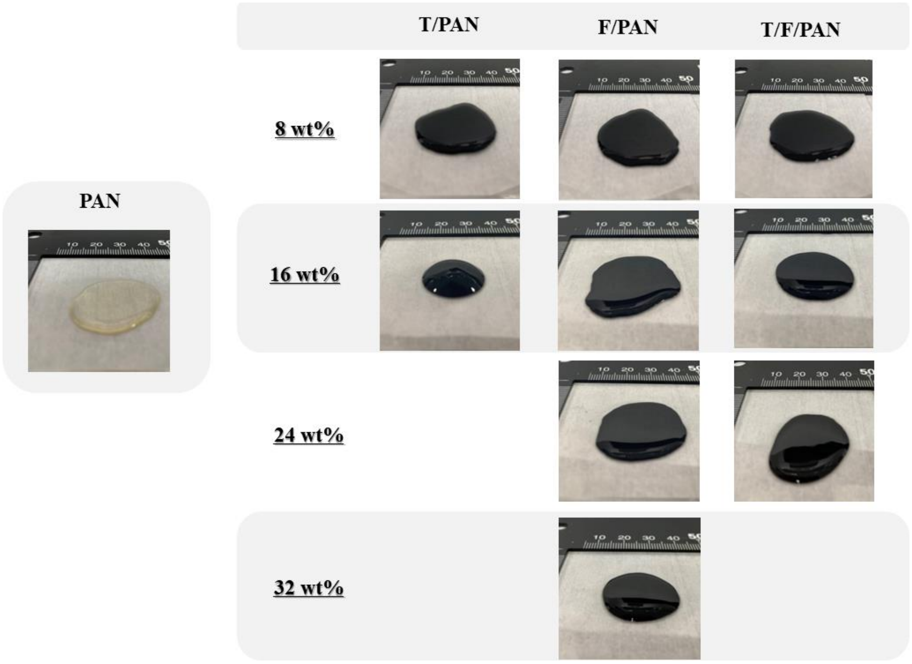

Figure S2. Visualized images of the PAN-based composite solution with various contents of carbon nanomaterials.
